# ATR-Chk1 signaling inhibition as a therapeutic strategy to enhance cisplatin chemosensitivity in urothelial bladder cancer

**DOI:** 10.18632/oncotarget.6482

**Published:** 2015-12-05

**Authors:** Ching-Chia Li, Juan-Cheng Yang, Mei-Chin Lu, Chia-Lin Lee, Chieh-Yu Peng, Wei-Yu Hsu, Yun-Hao Dai, Fang-Rong Chang, Da-Yong Zhang, Wen-Jeng Wu, Yang-Chang Wu

**Affiliations:** ^1^ Department of Urology, Kaohsiung Municipal Ta-Tung Hospital, Kaohsiung, Taiwan; ^2^ Department of Urology, Faculty of Medicine, College of Medicine, Kaohsiung Medical University, Kaohsiung, Taiwan; ^3^ Graduate Institute of Medicine, College of Medicine, Kaohsiung Medical University, Kaohsiung, Taiwan; ^4^ School of Pharmacy, College of Pharmacy, China Medical University, Taichung, Taiwan; ^5^ Chinese Medicine Research and Development Center, China Medical University Hospital, Taichung, Taiwan; ^6^ Graduate Institute of Marine Biotechnology, National Dong Hwa University, Pingtung, Taiwan; ^7^ Graduate Institute of Natural Products, Kaohsiung Medical University, Kaohsiung, Taiwan; ^8^ Center of Drug Discovery, College of Pharmacy, China Pharmaceutical University, Nanjing, China; ^9^ Department of Urology, Kaohsiung Municipal Hsiao-Kang Hospital, Kaohsiung, Taiwan; ^10^ Center of Molecular Medicine, China Medical University Hospital, Taichung, Taiwan

**Keywords:** bladder cancer, ATR-Chk1, natural products

## Abstract

DNA damage responses contribute to cisplatin resistance; however, therapeutic strategies to overcome cisplatin resistance have not yet been established. Here, we demonstrate that inhibition of ATR-Chk1 pathway with the potent inhibitor WYC0209 sensitizes bladder cancer cells to cisplatin. In the clinical microarray profile, high ATR expression is associated with poor prognosis in bladder cancer patients who receive chemotherapy. We show that pharmacological and genetic suppressing of ATR sensitized cells to cisplatin. Treatment with WYC0209 or siATR increased levels of cisplatin-DNA adducts, concomitant with decreased levels of p-glycoprotein expression. Additionally, Combinations of cisplatin and WYC0209 show synergistic activity against bladder cancer. Ultimately, WYC0209 enhanced the anti-tumor effects of cisplatin and suppressed p-glycoprotein expression in bladder cancer xenografts. These results indicate that inhibiting ATR-Chk1 activation with WYC0209 suppresses p-glycoprotein expression and increases cisplatin activity in bladder cancer. Our findings collectively suggest that ATR-Chk1 is a target for improving the efficacy of cisplatin in bladder cancer.

## INTRODUCTION

Urothelial carcinoma (UC) is a common malignant type of bladder cancer in the developed world. Bladder cancer is the fourth leading cause of cancer in men, accounting for 7% of all cancer cases and 4% of all cancer deaths [1]. Despite the surgical treatment of transurethral resection of the bladder tumor (TURBT), distant recurrences occur in many patients after primary treatment. The incidence of bladder recurrence within 5 years can be up to 20% to 75% worldwide [2]. From a clinical point of view, muscle-invasive bladder cancers have been associated with progressive disease with a poor prognosis, and treatment options have become limited [3]. Presently, cisplatin-based therapy is considered the standard-of-care for muscle-invasive bladder cancer [4]. Although cisplatin-based chemotherapy has improved the clinical outcome of patients with muscle-invasive bladder cancer, the major challenge of treatment remains cisplatin resistance [5]. Patients treated with cisplatin-based chemotherapy still have a poor outcome, and the therapeutic efficacy of cisplatin is limited, suggesting that some mechanisms remain unclear [3, 5]. DNA damage responses mediated through the ATR-Chk1 pathway are important factors for a therapeutic response and, therefore, are targets for new drug development [6-8]. However, the role of Chk1/2 signaling in the regulation of the cisplatin response in bladder cancer has largely been unexplored. Although DNA repair is important to cisplatin resistance, other mechanisms are involved. For example, substantial attention has been given to ATP-binding cassette (ABC) transporters, such as p-glycoprotein (also known as MDR1), which is commonly overexpressed in cancers [9, 10]. High p-glycoprotein expression was shown to correlate with a poor prognosis in bladder cancer patients after cisplatin-based adjuvant chemotherapy [11]. Interestingly, recent studies have shown that repressing p-glycoprotein via gene-silencing strategies is able to enhance the effects of cisplatin in hepatocellular carcinoma [12].

We and others have reported that the inhibition of ATR-Chk1 pathways could sensitize cancer cells to cisplatin treatment [13-15]. Although a partial response to the Chk1 inhibitor LY 2603618 was observed in a phase I clinical trial, the effect was moderate [16]. Recent approaches to the combination of these ATR-Chk1 inhibitors with chemotherapy have been evaluated in preclinical and clinical studies [17, 18]. However, how these combinations sensitize cancer cells to cisplatin therapy and whether these drug combinations are effective in clinical practice are unknown. Despite these potential strategies, there remains no effective therapy currently available for the treatment of bladder tumors expressing p-glycoprotein. Recent studies have revealed the inhibitory effects of flavonoid compounds on p-glycoprotein that are likely due, in part, to the multiple targets affected by its polyphenol structure [19]. Additionally, flavonoids can act as the core structure for designing modulators against p-glycoprotein activity [20]. This observation has led to the options for developing new anti-cancer agents. Therefore, we used a xenograft model to demonstrate that the flavonoid derivative WYC0209, when used in combination with cisplatin, might also have significant therapeutic activity.

Because multiple mechanisms may be responsible for the response to cisplatin treatment, the strategy that additional drug combinations will lead to the improvement of the therapeutic response is an important question in the development of new agents to enhance cisplatin activity. So far, the treatment of muscle-invasive bladder cancer with cisplatin remains a major challenge in developing effective drug combination strategies. We postulated that therapeutic targets for enhancing the effects of cisplatin may offer new opportunities for intervention.

In this study, we sought to identify therapeutic agents to enhance the sensitivity of cisplatin in bladder cancer. Here, we reported that the activity of cisplatin can be pharmacologically enhanced by WYC0209. Unexpectedly, we have found that WYC0209 suppressed the levels of p-glycoprotein and increased the levels of cisplatin-DNA adducts, triggering significant DNA damage and cell death. These results indicate that WYC0209 can suppress p-glycoprotein expression and serve as a potential lead for combating cisplatin resistance.

## RESULTS

### WYC02 and WYC0209 are anti-cancer agents that induce cell death in human bladder cancer cells

**Figure 1:**
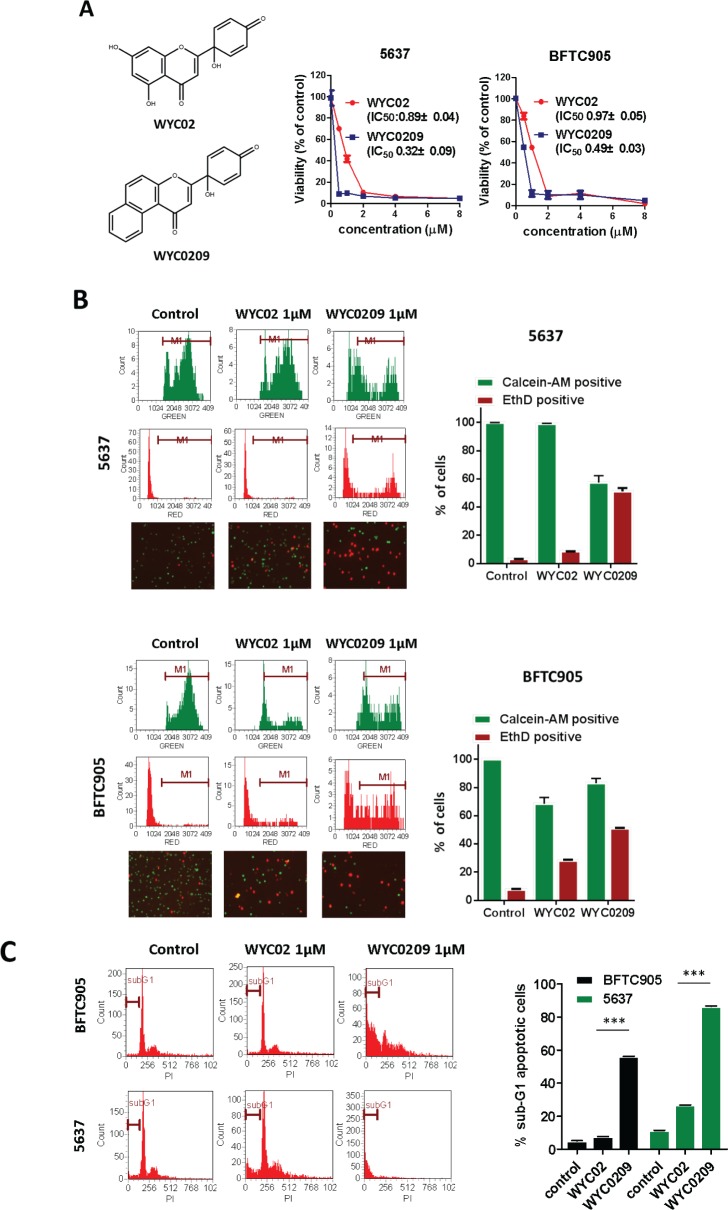
Effects of WYC02 and WYC0209 in human bladder cancer cell lines BFTC 905 and 5637 Cells were treated with WYC02 or WYC0209 for 3 days. **A.** Chemical structure of WYC02 and WYC0209 (left). Cell viability was determined by MTT assay (right). **B.** Cells were treated with WYC02 or WYC0209 at the concentration of 1μM for 2 days. Cytotoxicity and viability was determined by the Live/Dead cell viability assay. **C**. The percentage of apoptotic cells were determined by the proportion of sub-G1 cells. Cells were treated with WYC02 or WYC0209 at the concentration of 1μM for 2 days, and then stained with propidium iodide. The sub-G1 phase were analyzed by flow cytometry. Data represent mean±SEM of three replication.

Previously, we found that the natural product protoapigenone WYC02 is a potent anti-cancer agent using cell-based screening [21]. WYC02 inhibited cancer cell proliferation and increased cell death through the induction of ROS-mediated DNA damage and the activation of MAPK signaling pathways [22, 23]. Although these compounds showed growth inhibition in various cancer cell lines [21], their activity in bladder cancer has remained unknown. As shown in Figure 1A, the inhibitory activity of WYC02 and WYC0209 on cell viability in BFTC 905 and 5637 cells was examined. After treatment, WYC0209 robustly inhibited the viability of bladder cancer cells with an inhibition of cell viability (IC50) value of 0.49±0.03 μM and 0.32±0.09 μM in BFTC 905 and 5637 cells, respectively (Figure 1A). Notably, the activity of WYC0209 was 2-fold higher than that of WYC02 (IC50: 0.97±0.05 μM in BFTC 905 cells; 0.89±0.04 μM in 5637 cells). We next examined the ratio of death and viability using the live/dead assay. Cell viability was measured by the detection of the calcein-AM hydrolysis product calcein, which is an indicator of esterase activity, and cell death was measured by the detection of the EthD dye, which is an indicator of the damage of the plasma membrane. We found that WYC02 and WYC0209 potently affect cell viability and death as shown by calcein-AM activity and EthD staining (Figure 1B). At 1 μM, however, WYC02 did not induce cell death in BFTC 905 cells after a 48-h treatment, as revealed by the induction of 13.67±0.85% EthD-positive cells, but significantly increased cell death in 5637 cells (Figure 1B). Consistent with the viability results, the number of EthD-positive cells from WYC0209-treated cells was 2-fold higher in BFTC 905 and 5637 cells than in WYC02-treated cells. Similarly, the cell line 5637 exhibited higher sensitivity than BFTC 905 cells to WYC0209 (Figure 1B). The induction of cell death was further confirmed through the analysis of the cell cycle distribution. WYC0209 triggered an increase in the fraction of cells in sub-G1 up to 56.3±0.04% and 86.43±0.26% in BFTC 905 and 5637 cells, respectively (Figure 1C). By contrast, WYC02 induced a slight increase in the sub-G1 fraction in 5637 cells.

### High ATR expression correlates with poor patient outcome

**Figure 2:**
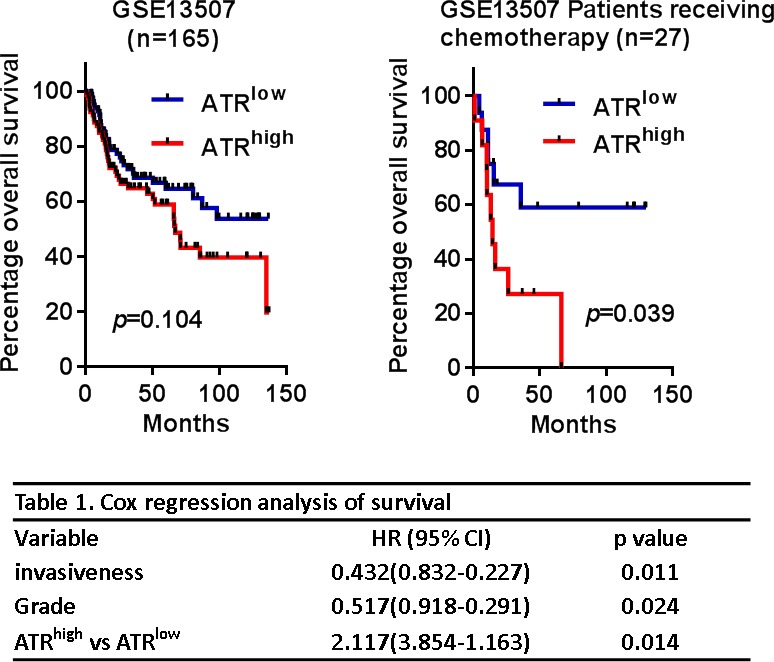
High ATR expression was associated with poor prognosis **A**. Kaplan-Meier analyses of all bladder cancer patients (*n* = 165) and patients receiving chemotherapy (*n* = 27) for overall survival stratified with ATR expression. **B**. Cox regression analyses of overall survival of bladder cancer patients with different risk factors.

The limited efficacy of cisplatin in bladder cancer treatment has been proposed to result from DNA Damage Response (DDR) pathway activation that can alter the sensitivity to DNA-damaging agents [9, 24]. The ATM/ATR signaling cascades are important pathways of the DNA damage response, and these signals lead to ATR-mediated Chk1 activation and ATM-mediated Chk2 activation, followed by the regulation of cell cycle progression and apoptosis [9]. Because cisplatin-based chemotherapy is the standard of care for muscle-invasive bladder cancer, it raises an interesting question of whether ATR expression correlates with patient outcome. To determine the clinical relevance of ATR's role in bladder cancer treatment, we assessed a gene expression dataset of 165 bladder cancers [GSE13507]. As shown in Figure 2A, high ATR expression was not significantly correlated with poor prognosis. However, it is interesting to note that patients receiving chemotherapy with high ATR expression in the tumors had a significant reduced survival time (*p* = 0.039). Cox hazard regression analysis revealed that high ATR expression was associated with higher risk of death (HR: 2.117; 95% CI: 3.854-1.163; *p* = 0.014). Taken together, these finding suggested that ATR might be targeted to improve therapeutic outcome in patients receiving cisplatin.

### ATR-Chk1 inhibition with WYC0209 improves cisplatin-induced DNA damage

**Figure 3:**
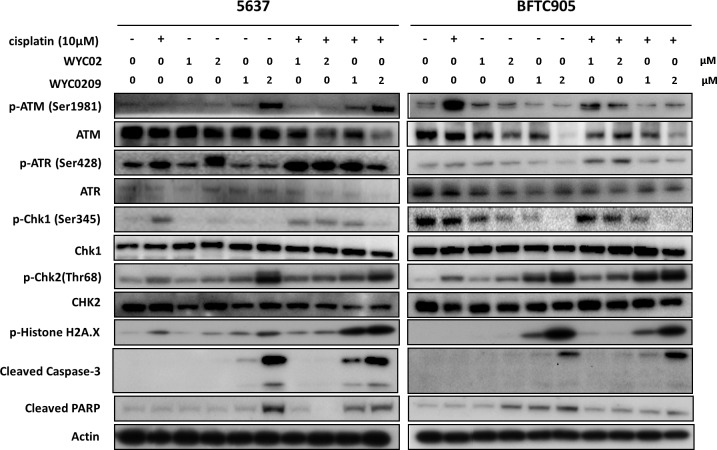
Western blot analysis for DNA Damage Responses (DDRs) and apoptosis pathway Cells were treated with WYC02, WYC0209, or combined with cisplatin (10 μM) for 24 h to determine ATR/ATM pathway, the levels of p-Histone H2A.X, cleaved caspase-3, and cleaved PARP-1.

As we and others have published previously [7, 13, 17], inhibition of the ATR-Chk1 pathway with selective inhibitors can sensitize cells to cisplatin. We then determined whether WYC0209 inhibited the activation of ATR-Chk1 selectively in bladder cancer cells; thus, strategies capable of inhibiting the DNA damage responses (DDRs) may be effective in muscle-invasive bladder cancer. As shown in Figure 3, treatment with WYC0209 inhibited cisplatin-induced ATR-Chk1 activation in bladder cancer cells. Notably, this activity was selective for Chk1, since the phosphorylation of Chk2 was elevated by treating with WYC0209. Note that WYC02 had little effect on the inhibition of Chk1 phosphorylation. To test whether these synthesized compounds, WYC02 and WYC0209, would enhance cisplatin-induced DNA damage, we initially treated bladder cancer cells with WYC02 or WYC0209 and measured the level of p-histone H2A.X. We also assessed the cleaved caspase 3 and cleaved PARP-1. Cisplatin had a modest effect on the induction of histone H2A phosphorylation at 10 μM. As predicted, the effects of these compounds on the cleaved caspase 3 and cleaved PARP-1 paralleled its effects on p-histone H2A.X induction in 5637 cells (Figure 3). Notably, treatment with WYC02 or WYC0209 had the moderate effect on the induction of cleaved caspase 3 and cleaved PARP-1 in BFTC 905 cells (Figure 3), suggesting that a distinct mechanism underlying these effects may decrease the activity of these compounds.

### WYC0209, but not WYC02, increases cisplatin-adduct DNA levels and inhibits p-glycoprotein expression and function

**Figure 4:**
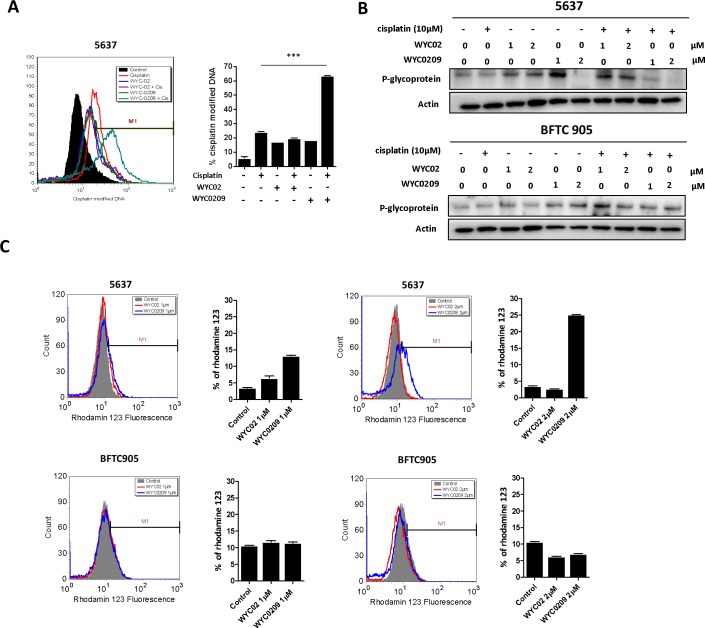
Effects of WYC02 and WYC0209 on the cisplatin-DNA adduct and the expression and activity of p-glycoprotein **A.** Effects of WYC02 and WYC0209 on cisplatin-DNA adduct. Cells were treated with WYC02 or WYC0209 combined with or without cisplatin (10 μM) for 24 h. The percentage of cisplatin-DNA adduct positive cells were measured by flow cytometry and are represented as mean±SEM of three replications. **B**. Membrane fraction analyses of p-glycoprotein expression. Cells were treated with WYC02, WYC0209, or combined with or without cisplatin (10 μM) for 24 h. The protein expressions were subjected to western blot analysis. **C.** The p-glycoprotein activity was assessed by the efflux of rhodamine-123 in BFTC 905 and 5637 cells. Cells treated with WYC02 or WYC0209 at the concentrations of 1 μM or 2 μM for 24 h. Cells were then treated with rhodamine-123 (20 μM) for 30 min. And cells were then refreshed in PBS. The accumulation of rhodamine-123 in cells was measured by flow cytometry.

Given our finding that ATR was associated with a poor prognosis and that WYC0209 can enhance cisplatin-induced DNA damage in bladder cancer cells promote us to test whether inhibition of ATR-Chk1 pathway with WYC0209 can alter cell susceptibility to cisplatin. Because both WYC02 and WYC0209 structure shares a similar pharmacological core, which contains 4-hydroxy-2,5-cyclohexadien-1-one moiety that exhibits multiple biological and pharmacological effects [25, 26], the mechanism underlying the WYC compound-mediated effects in bladder cancer cells remains unclear. We assessed the levels of cisplatin-DNA adducts, major determinants of cisplatin on-target effects. As shown in Figure 4A, cisplatin adduct levels were elevated in bladder cancer cells when cisplatin and WYC0209 were combined. The cisplatin-modified DNA-positive (cisplatin-DNA^+^) cells were increased from 24.11±0.39% to 63.53±0.21% in 5637 cells when cisplatin treatment was combined with WYC0209. However, unexpectedly, WYC02 did not increase the levels of cisplatin-DNA^+^ cells (Figure 4A). We conclude that WYC0209 is more effective than its parental compound WYC02 in enhancing the effects of cisplatin in bladder cancer.

Our finding of increased cisplatin activity in WYC0209-treated cells prompted us to investigate how WYC0209 enhances cisplatin activity. Because ABC transporters are thought to play a critical role in reducing the levels cellular chemotherapeutic drugs [9, 10], we assessed the levels of p-glycoprotein after combined WYC0209/cisplatin treatment. Analysis of protein expression in the membrane fractions using immunoblotting revealed that p-glycoprotein was suppressed by treatment with WYC0209 in 5637 cells (Figure 4B). Having demonstrated that WYC0209 effectively inhibited the levels of p-glycoprotein, we investigated the functional activity of p-glycoprotein using the rhodamine 123 fluorescent dye, a p-glycoprotein substrate. We assessed the efflux of rhodamine 123 as shown by FACS analysis. We analyzed both rhodamine 123-positive cells and the mean fluorescence intensity values after 24-h exposure to WYC02 or WYC0209. Analysis of FACS histograms showed that the accumulation of rhodamine 123 in cells was increased after WYC0209 treatment at the doses of 1 μM and 2 μM in 5637 cells (Figure 4C). However, consistent with the expression level of p-glycoprotein results showing that BFTC 905 cells were resistant to WYC0209, the efflux of rhodamine 123 showed no significant difference in the mean fluorescence intensity values after WYC02 or WYC0209 treatment in BFTC 905 cells (Figure 4C). In 5637 cells, WYC0209-treated cells exhibited a significant increase in the intracellular rhodamine 123 levels compared with WYC02-treated cells and control cells. Similarly, following WYC0209 treatment, approximately 13% and 25% of WYC02-treated cells showed high rhodamine 123 levels at the doses of 1 μM and 2 μM compared with WYC02 treatment or control (Figure 4C). These results demonstrated that WYC0209 can attenuate p-glycoprotein activity and expression.

**Figure 5:**
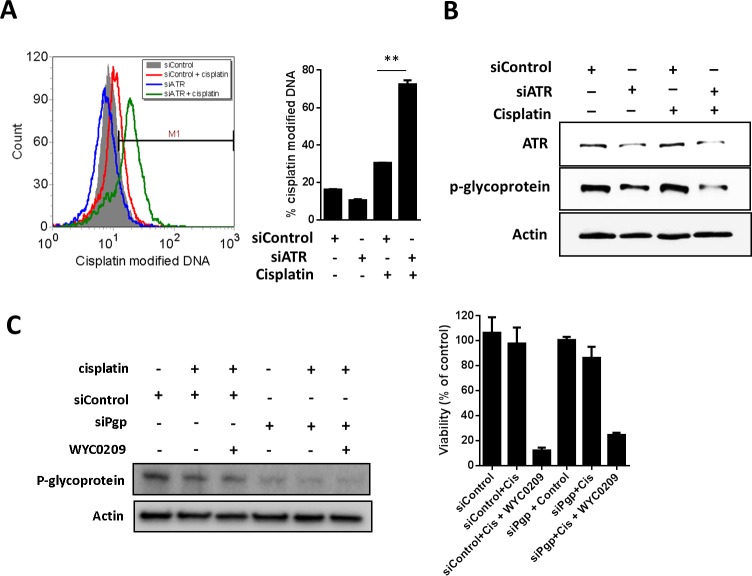
Knockdown of ATR or p-glycoprotein with siRNA increase the cisplatin-DNA adduct in 5637 cells Effects of siATR knockdown on **A.** cisplatin-DNA adduct and **B.** ATR and p-glycoprotein expression. Cells were treated with siATR or siControl combined with or without cisplatin (10 μM) for 24 h. Effects of siPgp knockdown on **C**. p-glycoprotein expression and viability. Cells were treated with siATR or siControl combined with or without cisplatin (10 μM) and WYC0209 (1 μM) for 24 h to assess the p-glycoprotein expression and for 48 h to assess the cell viability.

**Figure 6:**
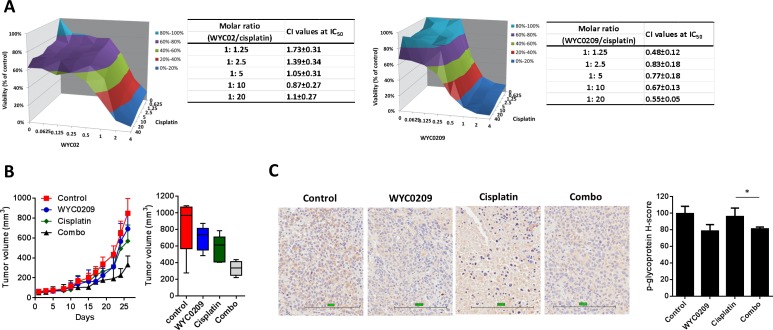
WYC0209 synergized with cisplatin and suppressed p-glycoprotein expression in xenograft animal model **A**. Synergistic effect of WYCs and cisplatin in 5637 bladder cancer cells [X-axis: WYC02 or WYC0209 (μM); Y-axis: cisplatin; Z-axis: Cell viability (%)]. Combination index (CI) values of WYCs/cisplatin combination were calculated by using CalcuSyn. **B**. *In vivo* antitumor effects of WYC0209 and WYC0209/cisplatin combination (Combo) were evaluated in 5637 xenografts. Boxplot of final tumor volumes. **C**. Representative IHC for p-glycoprotein expression in 5637 xenografs. Scale bar, 200 μm for micrograph. H-score for p-glycoprotein staining represents the levels of p-glycoprotein expressions. Data are represented as mean mean±SEM.

Since inhibition of ATR appears to sensitize tumors to cisplatin-induced cell death [15], the elevated cisplatin-DNA adducts evident here might be either an indirect off-target effect due to inhibition of p-glycoprotein or an indirect on-target effect on the inhibition of ATR-Chk1 pathway. To distinguish between two possibilities, we examined whether the increased cisplatin-DNA^+^ cells is a direct effect of ATR knockdown. Knockdown of ATR using siRNA resulted in a significant increased cisplatin-DNA^+^ cells up to 72.46±2.11% at 10 μM cisplatin treatment compared with cells transfected with siControl (30.57±0.01%; Figure 5A), demonstrating that the ability to increase cisplatin-DNA adducts is a direct effect from inhibition of ATR expression. While the increased cisplatin-DNA adducts is likely to reflect the downregulation of p-glycoprotein after treatment with WYC0209, we speculated that the increased cisplatin-DNA adducts is associated with the downregulation of p-glycoprotein and the inhibition of ATR. Knockdown of ATR using siATR affected p-glycoprotein levels in cells (Figure 5B). Treatment with siATR in the presence of cisplatin decreased the expression of p-glycoprotein (Figure 5B). Next, to determine if p-glycoprotein has a functional role in cisplatin treatment, we knock down the expression of p-glycoprotein using siRNA to test the response to cisplatin. As shown in Figure 5C, p-glycoprotein knockdown slightly enhance the activity of cisplatin. Additionally, the data showed that p-glycoprotein knockdown did not enhance the activity of WYC0209 and cisplatin combination (Figure 5C). Because expression of p-glycoprotein was not completely inhibited, we still cannot rule out the effect of ATR inhibition to DDRs in response to cisplatin. Together, these findings indicated that the efficacy of cisplatin could be improved, at least in part, by inhibition of ATR-Chk1 pathway. We hypothesize that combination of cisplatin plus WYC0209 could enhance cisplatin-induced cell death and that this combination might result in synergism. Therefore, the effects of WYC02 or WYC0209 combined with cisplatin were evaluated by using values of combination index (CI). As shown in Figure 6A, the interaction between WYC0209 and cisplatin was synergistic, whereas combination between WYC02 and cisplatin exhibited the addictive interaction. At 50% inhibitory effects, CI values for WYC0209/cisplatin were ranged from 0.83±0.18 to 0.48±0.12 (Figure 6A).

### WYC0209 reduces p-glycoprotein and inhibits tumor growth *in vivo*

Given the observation that inhibition of ATR suppresses the expression of p-glycoprotein, we hypothesize that ATR-Chk1 pathway was partly responsible for cisplatin resistance and that ATR-Chk1 pathway may be therapeutic targets for enhancing response to cisplatin. Thus, to address whether this combination strategy was effective *in vivo*, the nude mice bearing 5637 xenografts were treated with WYC0209 alone, cisplatin alone, and their combination. Mice treated with cisplatin or WYC0209 alone showed the moderate effect on the inhibition of tumor progression (Figure 6B). A combination treatment with WYC0209 and cisplatin robustly delayed the tumor growth in comparison to control group (Figure 6B). We then further test whether treatment with WYC0209 affects the expression of p-glycoprotein *in vivo*, we performed immunohistochemical experiments and semiquantitative histologic score (H-score) analysis. IHC analysis revealed that downregulation of p-glycoprotein was observed in xenografts treated with WYC0209 alone (median H-score = 78.6±3.8) or in combination with cisplatin (median H-score = 81.2±1.1) in comparison with vehicle control (median H-score = 99.6±4.4) or cisplatin alone (median H-score = 96±5.1; Figure 6C). Taken together, these data provide a possibility that inhibition of ATR-Chk1 with WYC0209 effectively enhance the activity of cisplatin, and cause a significant downregulation of p-glycoprotein.

## DISCUSSION

Resistance to cisplatin represents a major challenge for advanced bladder cancer. Earlier studies of ATR demonstrated a critical role for the DNA damage response following chemotherapy [24]. We have previously described a flavonoid class of anti-cancer agents comprising WYC02 and WYC0209 that effectively inhibited the ATR-Chk1 signaling cascade [13]. Consistently, the role of ATR in regulating DDR factors, such as Chk1 and Wee1, associated with DNA repair and apoptosis, is well established [6, 8]. We show here that inhibition of ATR-Chk1 pathway provides marked improvement in the response to cisplatin in bladder cancer.

Importantly, although ATR-Chk1 signaling components have been developed as therapeutic targets and the associated inhibitors are currently being entered into clinical trials for evaluation [27], a role for ATR in the progression of bladder cancer remains unknown as well as the exact mechanism by which ATR-Chk1 pathway affects the response to cisplatin. Interestingly, analysis of the dataset (GSE13507) showed that ATR mRNA expression correlated with overall survival in the patients treating with chemotherapy (Figure 2), suggesting that high ATR expression could serve as a marker for sensitivity to cisplatin in patients with bladder cancer. We propose the hypothesis that if cancer cells rely on ATR signaling for DNA repair, they are susceptible to genotoxic agents at least due in part to defects in DNA repair functions. Our findings showed that cisplatin-induced histone H2A.X phosphorylation on Ser 139 was elevated upon exposure to WYC02 or WYC0209. Similar results have also been observed with other inhibitors of ATR [7, 17]. These findings provide a combination strategy for bladder cancer with defects in DNA repair signaling.

Dysregulated DNA repair signaling appears to contribute to chemoresistance [9, 24]. Based on our previous findings, the mechanism by which WYC0209 mediates chemosensitization appears to involve the ATR-related homologous recombination repair process [13]. Mechanistic studies have found that the inhibition of Chk1 activation, which functions downstream of ATR, triggers synergistic effects with DNA-damaging agents [17], indicating an approach for cisplatin-based therapies. Notably, targeting DDR-related pathways has been proven to have clinical benefits for patient outcomes [28]. However, whether these findings can be extended to advanced bladder cancer is not fully understood. A combination with cisplatin and WYC0209 was sufficient to induce significant H2A.X phosphorylation (Figure 3). Notably, WYC0209, but not WYC02, effectively sensitized cells to cisplatin, as evidenced by elevated DNA-cisplatin adducts (Figure 4A). The data provided in the present study will undoubtedly provide insight into the treatment of advanced bladder cancer.

Additionally, ATR knockdown with siRNA resulted in an increase in cisplatin-DNA adducts (Figure 5A). These findings indicate the possibility that suppressing ATR-Chk1 signaling with WYC0209 enhances cisplatin activity through elevated DNA adducts and may synergize with other genotoxic drugs. Our previous findings demonstrated that MAP kinase, particularly p38, plays a critical role in WYC02-mediated effects [22]. Consistent with this, it has been reported that ATR is required for p38MAPK/MK2 activation and consequently is important for cell survival following drug-induced DNA damage in p53-deficient cells [29]. Therefore, the mechanism by which WYC0209 mediates enhancement of cisplatin activity is an important question for the current drug combination.

Cisplatin-induced p-glycoprotein activity has been well studied [10]. Overexpression of p-glycoprotein has been implicated in resistance through drug efflux, endowing cancer cells with the ability to survive even a high dose of cisplatin and consequently limiting the therapeutic outcome. These results highlight the potential mechanism to identify new agents involved in indirectly regulating the level of p-glycoprotein rather than directly inhibiting the activity of p-glycoprotein. Intriguingly, we showed that WYC0209 can suppress the expression of p-glycoprotein in cell membrane (Figure 4B). Drug transporters have been considered an important mechanism conferring pre-target cisplatin resistance through the efflux of cisplatin [9]. Notably, the downregulation of p-glycoprotein by WYC0209 or siATR was observed, and is accompanied by increased levels of cisplatin-DNA adducts (Figure 4 and Figure 5). We propose that the response to cisplatin could be enhanced through a combination treatment with WYC0209 and cisplatin. Therefore, we suggest two hypotheses for the enhancement of cisplatin activity. First, ATR-Chk1 signaling is engaged in DNA repair, and inhibition of ATR-Chk1 pathway may affect the repair of cisplatin-DNA adducts. Our laboratory has previously demonstrated that homologous recombination repair is inhibited by WYCs, and the process may be important to the functions that render cancer cells more susceptible to cisplatin [13]. Second, inhibition of ATR-Chk1 activation may impact the levels of p-glycoprotein through a distinct mechanism that causes an increase in cisplatin in cells. The data reported here show that cisplatin in combination with WYC0209 has a synergistic anticancer effect (Figure 6A). Additionally, a combination therapy with cisplatin and WYC0209 has a significant improvement of anti-tumor activity (Figure 6B).

Taken together, our studies reveal a critical role for ATR-Chk1 signaling in mediating cisplatin resistance. We have identified a potential small-molecule inhibitor of ATR-Chk1 signaling as an attractive treatment for advanced bladder cancer. In combination with cisplatin, WYC0209 appears to selectively inhibit the ATR-Chk1 pathway, resulting in a significant enhancement of cisplatin activity. Similarly, others have also noted that ATR-Chk1 pathway inhibitors sensitize cancer cells to cisplatin independent of ATR kinase activity [17]. Given that ATR knockdown suppresses p-glycoprotein expression, cisplatin and WYC0209 combination may be useful for treating bladder cancer. In this study, we propose a strategy to improve cisplatin efficacy by combining the drug with an ATR-Chk1 pathway inhibitor.

## MATERIALS AND METHODS

### Materials

WYC02 (protoapigenone) and WYC0209 were chemically synthesized as described previously (Figure 1A) [30]. All chemical compounds were purchased from Sigma-Aldrich and Acros Organics.

### Cell cultures and drug treatments

The human bladder cancer cell lines BFTC 905 and 5637 were purchased from Bioresource Collection and Research Center (Hsinchu, Taiwan). For BFTC 905 cells, RPMI 1640 medium (Hyclone) was supplemented with 15% FBS and 1.5 g/L sodium bicarbonate. 5637 cells were cultured with RPMI 1640 medium supplemented with 15% FBS (GIBCO), 2 mM L-glutamine, and 1 mM sodium pyruvate. Cells were grown at 37°C in a 5% CO_2_ atmosphere. The compounds WYC02 and WYC0209 were dissolved in DMSO to prepare stock solutions that were stored at −80°C. The compounds were diluted in medium, and the final DMSO concentrations were less than 0.2%.

### Cell viability assay

BFTC 905 and 5637 bladder cancer cells were seeded in 96-well plates at a density of 10^4^ cells/well and incubated in culture medium for stabilization. The culture medium was then replaced with medium containing the indicated treatment for 3 days. Cell viability was assessed using the MTT assay as described previously [31]. Briefly, cells were added to an MTT working solution (3 mg/ml) at 37°C. After 1 h, the MTT solution was removed, and the insoluble formazan was dissolved by DMSO. The absorbance at 550 nm was read using an ELISA reader. The cell viability was calculated using the following formula: (A _sample_−A _blank_)/(A _control_−A _blank_)×100%. The cells treated with the vehicle DMSO served as the control group.

### Live/dead assay

Cellular viability and cytotoxicity were evaluated using the Live/Dead assay kit (Invitrogen) as evident by fluorescence intensity (calcein AM fluorescence in live cells and ethidium homodimer-1 (EthD) in dead cells). Briefly, the bladder cancer cells were washed with PBS to remove the culture medium. The cells were incubated with 2 μM calcein AM and 4 μM EthD-1 in 100 μL of working solution for approximately 40 min. Following incubation, cells were washed with PBS. For measurement, fluorescence was detected and imaged using Tali image-based cytometry (Invitrogen).

### Propidium iodide staining

Late-phase apoptosis was assessed by cell cycle sub-G1 analysis. Briefly, the cells were treated with WYC02 or WYC0209. The cells were washed with PBS and then fixed with 70% ethanol and stained with staining buffer (0.5% TritonX-100, 50 mg/ml PI, and 1 μg/ml RNase) at 37°C for 30 min. Apoptosis was measured by the proportion of the sub-G1 population using flow cytometry and the ModFit program (BD Biosciences).

### Gene expression analysis

The microarray dataset and clinical information was downloaded from Gene Expression Omnibus (GSE13507, http://www.ncbi.nlm.nih.gov/geo/query/acc.cgi?acc=GSE13507) [32]. To normalize the intensity values of each sample, the raw microarray dataset were processed using R and Bioconductor. The expression values for ATR were stratified as low or high ATR expression using the median value. The difference between low and high ATR expression was analyzed by Kaplan-Meier survival analysis determined by log-rank test. Cox regression analysis was performed to quantitate a hazard ratio (HR) and p value for each factor.

### Cisplatin-DNA adduct staining

The method used for assessing cisplatin adducts was described previously [33]. Briefly, cells treated with siRNA or WYCs were incubated with staining solution containing anti-cisplatin-modified DNA antibody (clone CP9/19; Abcam) and 100 mg/ml digitonin for 12 h. Cells were then incubated with secondary antibody for 2 h. The signals were analyzed using flow cytometry (BD Biosciences) and FCS Express v3.0 (De Novo Software).

### P-glycoprotein activity assay

The assessment of p-glycoprotein activity was detected by the efflux of rhodamine 123. Cells (10^5^) cells were treated with WYC02 or WYC0209 for 24 h. Additionally, cells were then treated with the p-glycoprotein substrate rhodamine 123 (20 μM) for 30 min. After treatment, cells were refreshed in culture medium for 2 h. Cells were then collected and washed with PBS. The levels of rhodamine 123 were analyzed by flow cytometry and FCS Express v3.0 (De Novo Software).

### siRNA knockdown

Cells were treated with siRNA (for ATR, siATR sequence: 5′- AAAUCAAGCAACAUCACGGAGGUUUG-3′; for p-glycoprotein, siPgp sequence: 5′-AAUGCAAUCACAGUUCUAAUUGCUG-3′; and for Scrambled control siRNA, siControl: 5′-UUCUCCGAACGUGUCACGUTT-3′), purchased from GeneDire X. For the transfection of siRNA, cells (10^6^) were seeded into 6-well plates and then were transfected with siRNA in diluted Lipofectamine RNAiMAX containing Opti-MEM Medium (Invitrogen). siControl nontargeting siRNA was used as the negative control.

### Immunoblotting and immunohistochemical (IHC) analyses

Immunoblotting was performed as described previously [13, 31]. Total cell lysates were lysed in RIPA buffer containing 100 μM sodium orthovanadate, 1 mM PMSF, and protease inhibitor cocktail. Additionally, membrane fractionations were performed using the Mem-PER plus membrane protein extraction kit (Pierce). The membrane proteins were separated according to the manufacturer's instructions. Protein concentrations were determined using the BCA Protein Assay Kit (Pierce). The proteins were separated by SDS-PAGE and then transferred to PVDF membranes. The levels of protein expression were detected using primary antibodies against p-Histone H2A.X (Cell Signaling), cleaved caspase-3 (Cell Signaling), cleaved PARP-1 (Cell Signaling), ATR (Abcam), ATM (Abcam), pSer428-ATR (Cell Signaling), pSer1981-ATM (GeneTex), pSer345-Chk1 (Cell Signaling), pThr68-Chk2 (Cell Signaling), Chk1 (Santa Cruz Biotechnology), Chk2 (Abcam), p-glycoprotein (GeneTex), and β-actin (GeneTex). The bands were detected using a chemiluminescent substrate (Millipore). The expression of p-glycoprotein was evaluated by immunohistochemistry (IHC) of xenograft tumor tissue. Antigen retrieval was performed in a pressure cooker for 20 min. The primary antibody against p-glycoprotein (Abcam) was applied at a dilution of 1:50 for 1 h. The tissue was incubated with Antibody Amplifier Quanto (Thermo) for 10 min and with HRP Polymer Quanto for 10 min. The slides were incubated with DAB Quanto Chromogen/substrate (Chromogen:substrate = 1:30) for 3 min. The slides were rinsed with water and were counterstained with hematoxylin for 2 min. The slides were dehydrated at 60°C for 10 min and were mounted for analysis. P-glycoprotein expression was determined using the semiquantitative H-score (0-300) obtained by scanning the slides and analyzing them using ImageScope (Aperio; Leica Biosystems).

### Xenograft studies

Xenograft experiments were performed at the China Medical University. All animal experiments were performed according to the Institutional Animal Care and Use Committee (IACUC) of China Medical University guidelines. The mice were fed sterilized food and autoclaved water. Five-week-old BALB/c nu-nu mice were purchased from the National Laboratory Animal and Research Center (Taipei, Taiwan) and maintained under pathogen-free conditions. Bladder cancer 5637 (5×10^6^) cells were resuspended in saline buffer and Matrigel (1:1) and then implanted s.c. into the right flank. Tumor growth was monitored daily. The tumor size was measured using calipers and determined by the following formula: volume (mm^3^) = length×width^2^/2. Once the tumor volume was greater than 100 mm^3^, the mice were segregated into four groups randomly (*n* = 5). The mice were intravenously injected with a). Control group: solvent (5% DMSO, 35% cremophor, and 60% saline); b). WYC0209 group: 2mg/Kg WYC0209 qw (135); c). Cisplatin group: 2 mg/kg cisplatin qwk; d). Combo group: 2 mg/kg cisplatin qwk and 2 mg/kg WYC0209 qw (135). The animals were sacrificed using carbon dioxide asphyxiation according to institution guidelines.

### Statistical analyses

The data are presented as the mean±SEM from three determinations. Significant differences were compared and analyzed by Student's *t* test. The synergistic effects of WYC0209 or WYC02 in combination with cisplatin were determined by the combination index (CI) using Calcusyn software (Biosoft, Ferguson, MO) [34]. For all experiments, the significance was defined as a p value less than 0.05 (*, *p* < 0.05; **, *p* < 0.01; ***, *p* < 0.001). Statistical analyses were analyzed using GraphPad Prism 5 and SPSS 12.0.
